# Under Heparin-Free Conditions Unsaturated Phospholipids Inhibit the Aggregation of 1N4R and 2N4R Tau

**DOI:** 10.1021/acs.jpclett.4c01718

**Published:** 2024-08-14

**Authors:** Abid Ali, Mikhail Matveyenka, Axell Rodriguez, Dmitry Kurouski

**Affiliations:** † Department of Biochemistry and Biophysics, Texas A&M University, College Station, Texas 77843, United States; ‡ Department of Biomedical Engineering, Texas A&M University, College Station, Texas 77843, United States

## Abstract

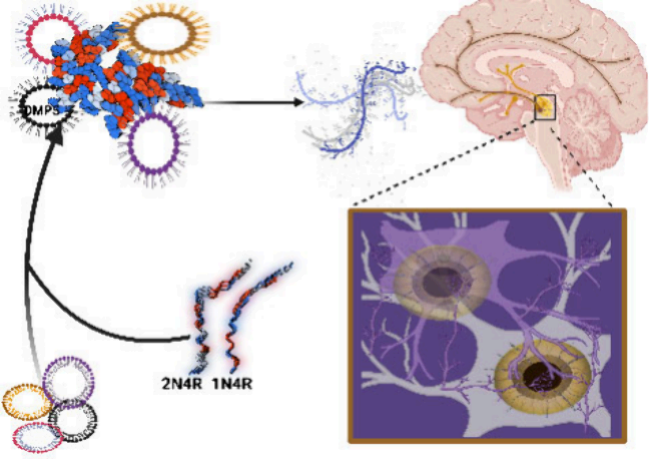

A progressive aggregation of Tau proteins in the brain is linked to both Alzheimer’s disease (AD) and various Tauopathies. This pathological process can be enhanced by several substances, including heparin. However, very little if anything is known about molecules that can inhibit the aggregation of Tau isoforms. In this study, we examined the effect of phosphatidylserines (PSs) with various lengths and saturations of fatty acids (FAs) on the aggregation properties of Tau isoforms with one (1N4R) and two (2N4R) N-terminal inserts that enhance binding of Tau to tubulin. We found that PS with unsaturated and short-length FAs inhibited Tau aggregation and drastically lowered the toxicity of Tau oligomers that were formed in the presence of such phospholipids. Such an effect was not observed for PS with fully saturated long-chain FAs. These results suggest that a short-chain irreversible disbalance between saturated and unsaturated lipids in the brain could be the trigger of Tau aggregation.

Microtubules in neurons are stabilized by several isoforms of Tau produced as a result of the alternative splicing of exons 2, 3, and 10.^1−4^ These isoforms can have one (1N4R) or two (2N4R) N terminal inserts that enhance the binding of Tau isoforms to the tubulin of microtubules.^5−8^ Numerous studies show that heparin can facilitate Tau aggregation, which results in the formation of toxic oligomers and fibrils.^9−11^ Such aggregates are found in neurofibrillary tangles (NFTs), intracellular formations found in patients diagnosed with Alzheimer’s disease (AD).^12,13^ These and other pieces of evidence indicate an important role of Tau aggregation in the onset and development of AD and other neurodegenerative pathologies such as Tauopathies.^12,14−17^

Ait-Bouziad and co-workers recently reported that phosphatidylserine (PS) could bind to 2N4R Tau and its K18 isoform.^13^ This anionic lipid under physiological conditions is primarily localized on the inner part of plasma membranes. Upon cell malfunctioning, the shortage of ATP prevents inner membrane localization of PS. This results in the increase of the concentration of PS on the exterior side of the membranes. Such cells are recognized and removed by macrophages. Using nuclear magnetic resonance (NMR) and cell toxicity assays, Ait-Bouziad found that Tau-PS oligomers formed in the presence of heparin were stabilized by strong electrostatic interactions between the charged amino acids residues of Tau and polar heads of PS.^13^ Such oligomers exerted substantially greater cell toxicity compared to 2N4R and K18 Tau aggregates formed in a lipid-free environment.^13^

Our group showed that aggregation properties of amyloidogenic proteins, including α-synuclein (α-Syn), insulin, lysozyme, and transthyretin (TTR), could be altered by the fatty acids (FAs) possessed by PS.^18−26^ For instance, PS with unsaturated FAs (1-palmitoyl-2-oleoyl-*sn*-glycero-3-phospho-l-serine (16:0–18:1, POPS) and 1,2-dioleoyl-*sn*-glycero-3-phospho-l-serine (18:1, DOPS)) accelerated the rate of α-Syn aggregation and increased toxicity of α-Syn fibrils.^20^ At the same time, the presence of PS with fully saturated FAs (1,2-distearyl-*sn*-glycero-3-phospho-l-serine (18:0, DSPS)) drastically lowered the toxicity of α-Syn fibrils.^20^ Frese and co-workers showed that PS with a very short FA (1,2-dimyristoyl-*sn*-glycero-3-phospho-l-serine (14:0, DMPS)) drastically lowered the stability of lysozyme, whereas DSPS, on the other hand, decelerated the rate of lysozyme aggregation.^27^ Furthermore, lysozyme fibrils formed in the presence of DMPS also exerted much higher cell toxicity compared to lysozyme:DSPS fibrils.^27^

In the current study, we investigated the effect of FAs in PS on the aggregation properties of 1N4R and 2N4R Tau in a heparin-free environment. We have chosen large unilamellar vesicles (LUVs) composed of PS with fully saturated FAs, 14:0 DMPS and 18:0 DSPS, as well as PS with unsaturated FAs, 18:1 DOPS and 16:0–18:1 POPS, Scheme 1. Using the thioflavin T assay, we investigated the aggregation kinetics of 1N4R and 2N4R Tau in the presence and absence of equimolar concentrations of the phospholipids. It is important to note that the vast majority of reported to date studies on Tau aggregation were performed in the presence of heparin, a negatively charged polysaccharide that facilitates Tau aggregation.^7,8,11,13−15,17,28^ However, the presence of this anionic molecule may not entirely represent physiological processes that occur in the brain upon AD and Tauopathies as well as obscure the clarification of protein–lipid interactions. Therefore, all performed experiments were made in a heparin-free environment.

**Scheme 1:**
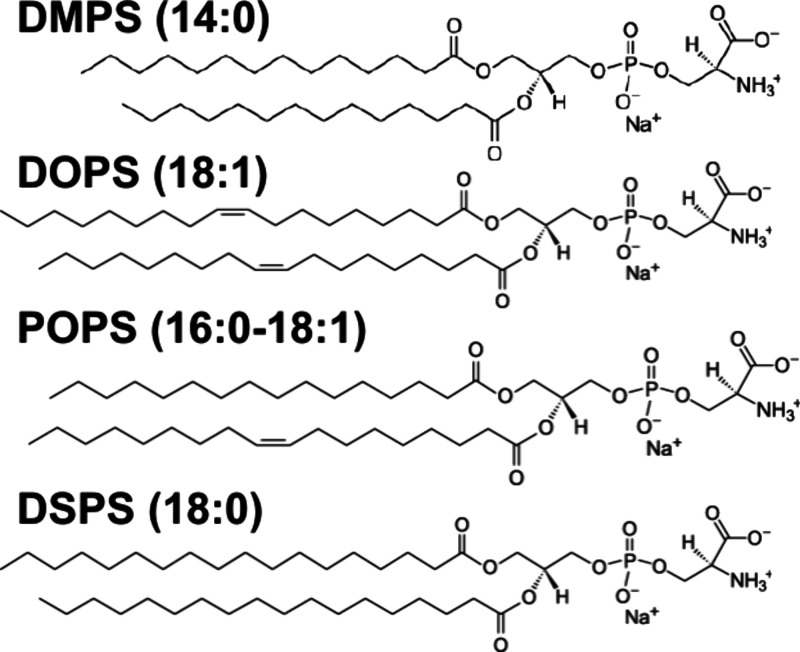
Molecular Structures of DMPS, DOPS, POPS, and DSPS

In the heparin- and lipid-free environment, 1N4R Tau aggregated with *t*_lag_ of 74.55 ± 4.25 h, Figure 1. The presence of DSPS at 1:5 molar ratio drastically increased the onset of 1N4R Tau aggregation to *t*_lag_ = 100 ± 0.1 h. DSPS also decelerated the rate of 1N4R Tau aggregation *t*_1/2_ from 90.66 ± 2.49 h (1N4R Tau) to 119 ± 0.98 h (1N4R Tau:DSPS). At the same time, we found no evidence of protein aggregation in the presence of DMPS, DOPS, and POPS. These results indicate that PS strongly altered Tau aggregation. Furthermore, long FA PS (DSPS) decelerated 1N4R Tau aggregation, whereas short FA PS (DMPS) and PS with unsaturated FAs (POPS and DOPS) completely inhibited protein aggregation.

**Figure 1:**
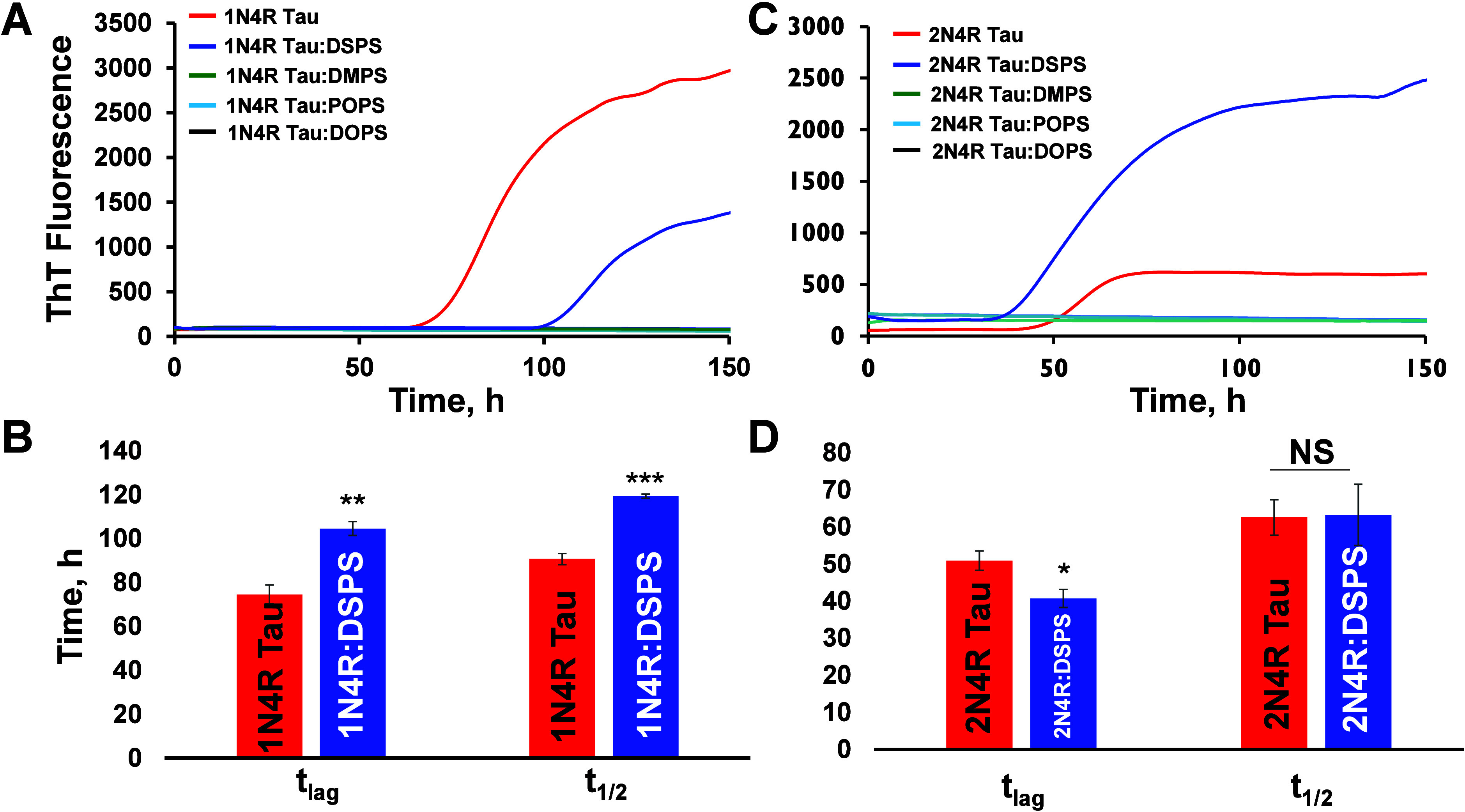
ThT kinetics of 1N4R (A) and 2N4R Tau (B) aggregation in the heparin-free environment in the absence of lipids and in the presence of DSPS, DMPS, DOPS, and POPS. Corresponding histograms of *t*_lag_ and *t*_1/2_ represent 10% and 50% increases in the ThT signals, respectively. According to one-way ANOVA, NS is nonsignificant difference, *P < 0.05, **P < 0.01, and ***P < 0.001.

In the heparin- and lipid-free environment, 2N4R Tau aggregated much faster than its shorter 1N4R Tau analogue, *t*_lag_ = 50 ± 2.61 h. We also found that DSPS (1:5) molar ratio accelerated rather than decelerated 2N4R Tau aggregation, *t*_lag_ = 40 ± 2.4 h, while no changes in the rate of protein aggregation *t*_1/2_ were observed. Similar to 1N4R, the aggregation of 2N4R Tau was completely inhibited by DMPS, DOPS, and POPS. Based on these results, we can conclude that DSPS has an opposite effect on 1N4R and 2N4R Tau aggregation, whereas POPS, DOPS, and DMPS strongly inhibit the aggregation of both Tau isoforms.

Morphological characterization of protein aggregates formed after 200 h of 1N4R and 2N4R Tau aggregation in heparin- and lipid-free environments revealed the presence of long fibrils that had ∼3–12 nm in height, Figure 2. We also found that, in the presence of DSPS, 1N4R Tau formed morphologically similar fibrils with heights ranging from 6 to 12 nm, Figure 2. With similar heights (3–12 nm), shorter fibrils were found in the 2N4R:DSPS sample. We also found a substantially larger number of spherical oligomers in this sample. No fibrils were found in 1N4R Tau:DMPS, DOPS, and POPS and 2N4R Tau:DMPS, DOPS, and POPS samples. Most of these samples possessed only small spherical oligomers that were 3–9 nm in height. Furthermore, we observed intact LUVs in nearly all of these samples.

**Figure 2:**
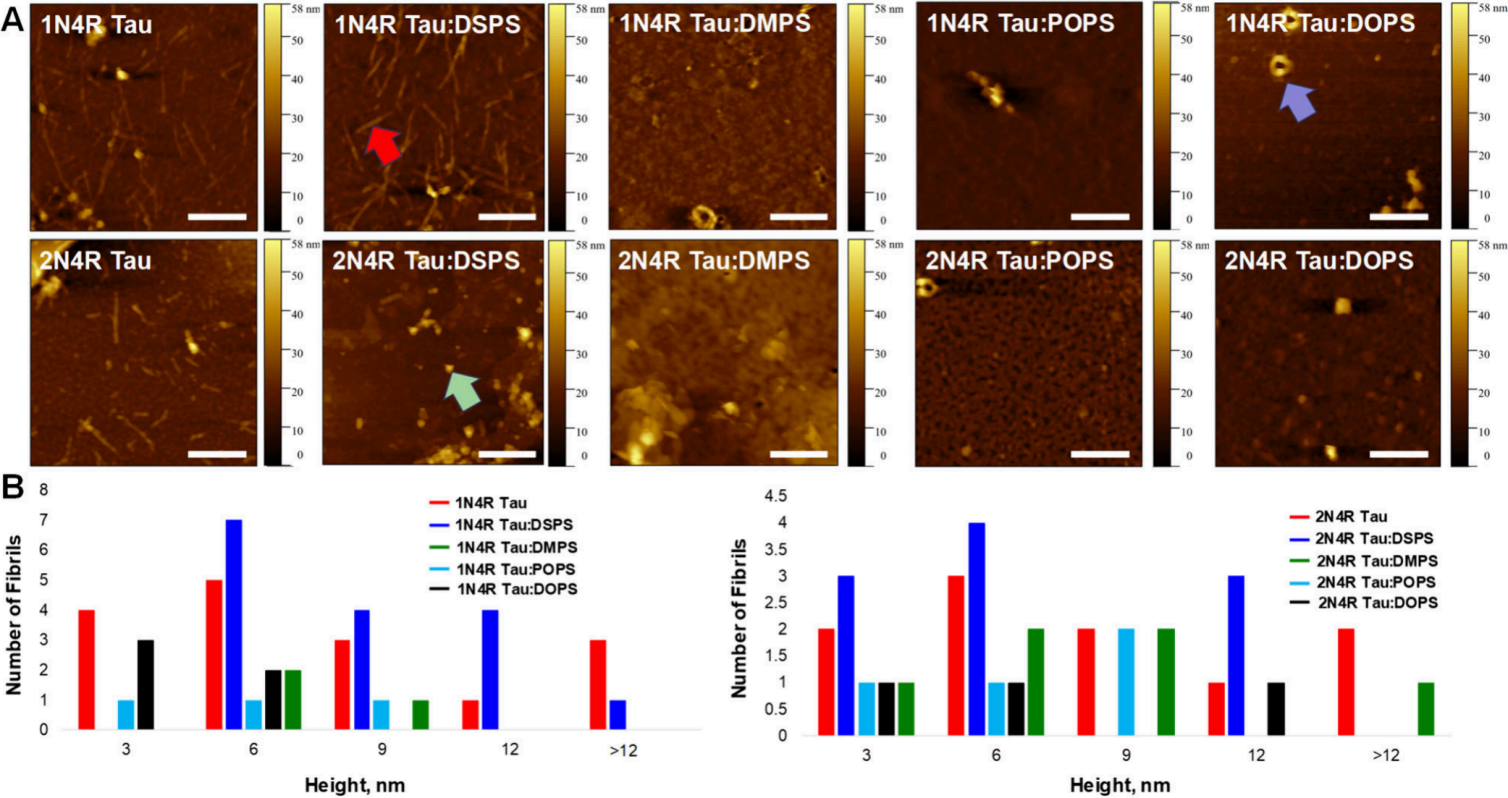
AFM images (A) of 1N4R (top row) and 2N4R Tau (bottom row) aggregates formed in the heparin-free environment with and without lipids. The scale bar is 500 nm. Fibrils and oligomers are shown by red and green arrows, respectively. LUV is highlighted with a purple arrow. Height profiles (B) of protein aggregates are shown in each sample.

Next, we utilized FTIR spectroscopy to examine the secondary structure of protein aggregates formed in a heparin-free environment with and without lipid LUVs. The acquired spectra exhibited amide I (1600–1700 cm^–1^)^29,30^ and II (1500–1570 cm^–1^)^31−33^ bands as well as CαH (1460 cm^–1^)^34^ vibration, Figure 3. We also found that protein aggregates that were formed in the presence of PS LUVs exhibited an intense band at ∼1730 cm^–1^ that originates from the C=O vibration of lipids.^24,35^ As expected, this band was not observed in the samples that did not contain LUVs (1N4R and 2N4R Tau). The amide I band is typically utilized to examine the secondary structure of protein aggregates. If centered around 1625 cm^–1^, protein samples are dominated by a parallel β-sheet, whereas its shift to 1645 cm^–1^ is indicative of an α-helix. Unordered protein exhibits amide I at ∼1660 cm^–1^, whereas the presence of an antiparallel β-sheet could be confirmed by the shift of amide I to 1695 cm^–1^.^31−33^

**Figure 3:**
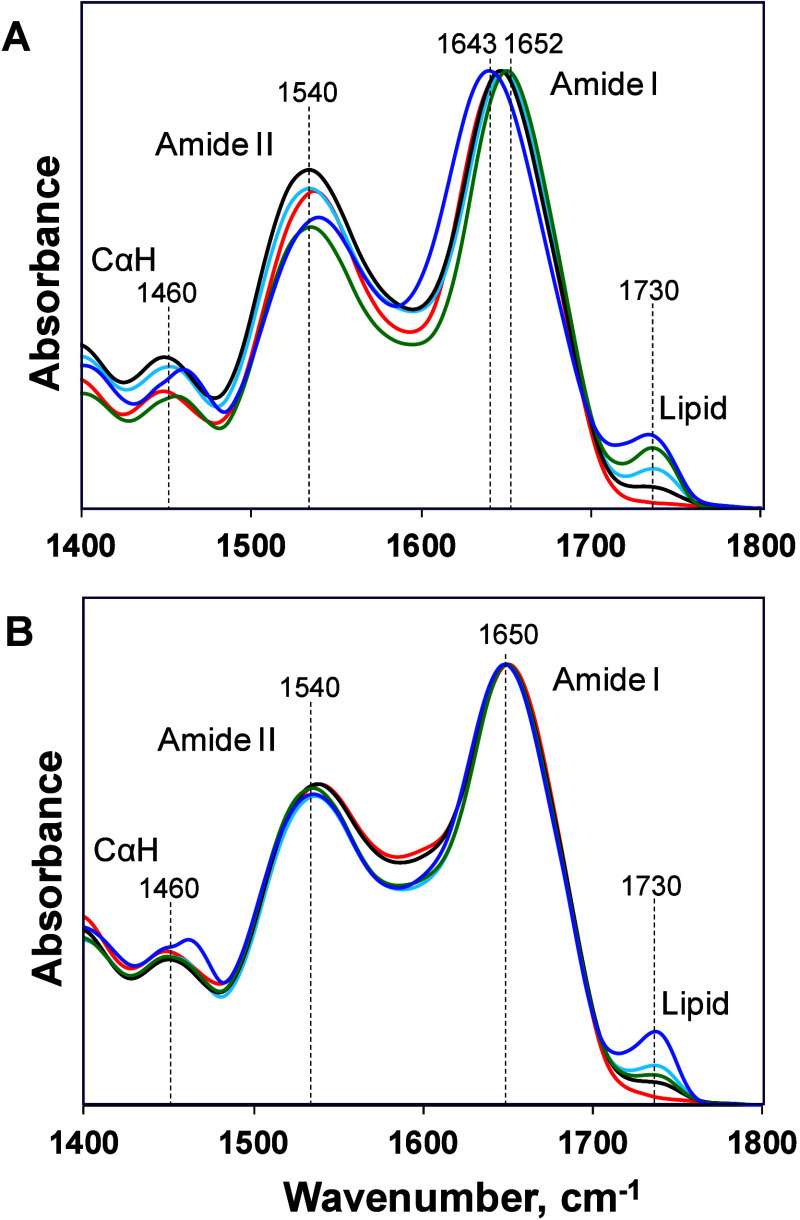
FTIR spectra of 1N4R (A) and 2N4R Tau (B) aggregation in a heparin-free environment in the absence of lipids (red) and in the presence of DSPS (blue), DMPS (green), DOPS (black), and POPS (light blue). Spectra are normalized on the amide I band.

All 1N4R Tau samples except 1N4R:DSPS exhibited amide I at around 1652 cm^–1^, whereas 2N4R Tau samples with and without lipids had amide I centered around 1650 cm^–1^. These surprising results indicate that 1N4R Tau, 1N4R:DSPS, 2N4R Tau, and 2N4R:DSPS are dominated by an α-helix rather than a β-sheet secondary structure, as one could expect based on the AFM images discussed above. These findings are further supported by CD spectra acquired from all analyzed samples, Figure S1. In addition to the minima around 220 nm, which indicate the presence of β-sheet secondary structure, acquired spectra exhibited small dips at ∼233–235 nm, characteristic for α-helix. We also found that amide I in the FTIR spectrum of 1N4R:DSPS was blue-shifted to 1643 cm^–1^. This shift indicates possible interactions between Tau protein and the lipid.^36^ We hypothesized that the dominance of the α-helical signal in FTIR spectra could be attributed to an unaggregated Tau protein that was present in the samples. To overcome this limitation of FTIR, we utilized nano-IR spectroscopy, also known as atomic force microscopy and infrared (AFM-IR) spectroscopy.

In AFM-IR, the metallized scanning probe can be positioned directly at the surface of the protein aggregate.^37,38^ Next, pulsed tunable light is used to induce thermal expansions that are reported by the scanning probe and converted into an IR spectrum.^39−43^ Thus, AFM-IR allows for overcoming the contribution of a background signal of unordered protein that is evident in FTIR, which probes the entire volume of analyzed samples.

AFM-IR spectra acquired from 1N4R Tau and 1N4R:DSPS fibrils exhibit an amide I band centered around 1625 cm^–1^, which indicates the predominance of a parallel β-sheet in their secondary structure, Figure 4A,B, Figures S2 and S3. It should be noted that lipids themselves did not exhibit substantial spectroscopic features in the amide I region of the IR spectra, Figure S4. Fitting of the amide I band in both spectra revealed a highly similar amount of the parallel β-sheet in their secondary structure in both 1N4R Tau and 1N4R:DSPS fibrils, Figure 4. It should be noted that individual AFM-IR spectra acquired from protein aggregates are shown in Figures S5 and S6. These protein aggregates also had ∼10% unordered protein and ∼30% antiparallel β-sheet in their structure. We also found that, in the AFM-IR spectra acquired from 1N4R:DOPS and 1N4R:DMPS oligomers, the amide I band was shifted to ∼1600 cm^–1^, which could be attributed to the vibration of amino acid side chains. The fitting of the amide I band in these spectra revealed a much larger amount of parallel β-sheet compared to 1N4R Tau and 1N4R:DSPS fibrils. We also found that the secondary structure of 1N4R:POPS oligomers was very similar to the secondary structure of 1N4R Tau and 1N4R:DSPS fibrils. However, we observed an intense lipid vibration in the AFM-IR spectra acquired from these aggregates. This indicates that POPS lipids could be present in their structure. These results indicate that DSPS and POPS did not alter the secondary structure of 1N4R Tau aggregates, while the presence of DOPS and DMPS resulted in the increase in the parallel β-sheet in the amyloid oligomers.

**Figure 4:**
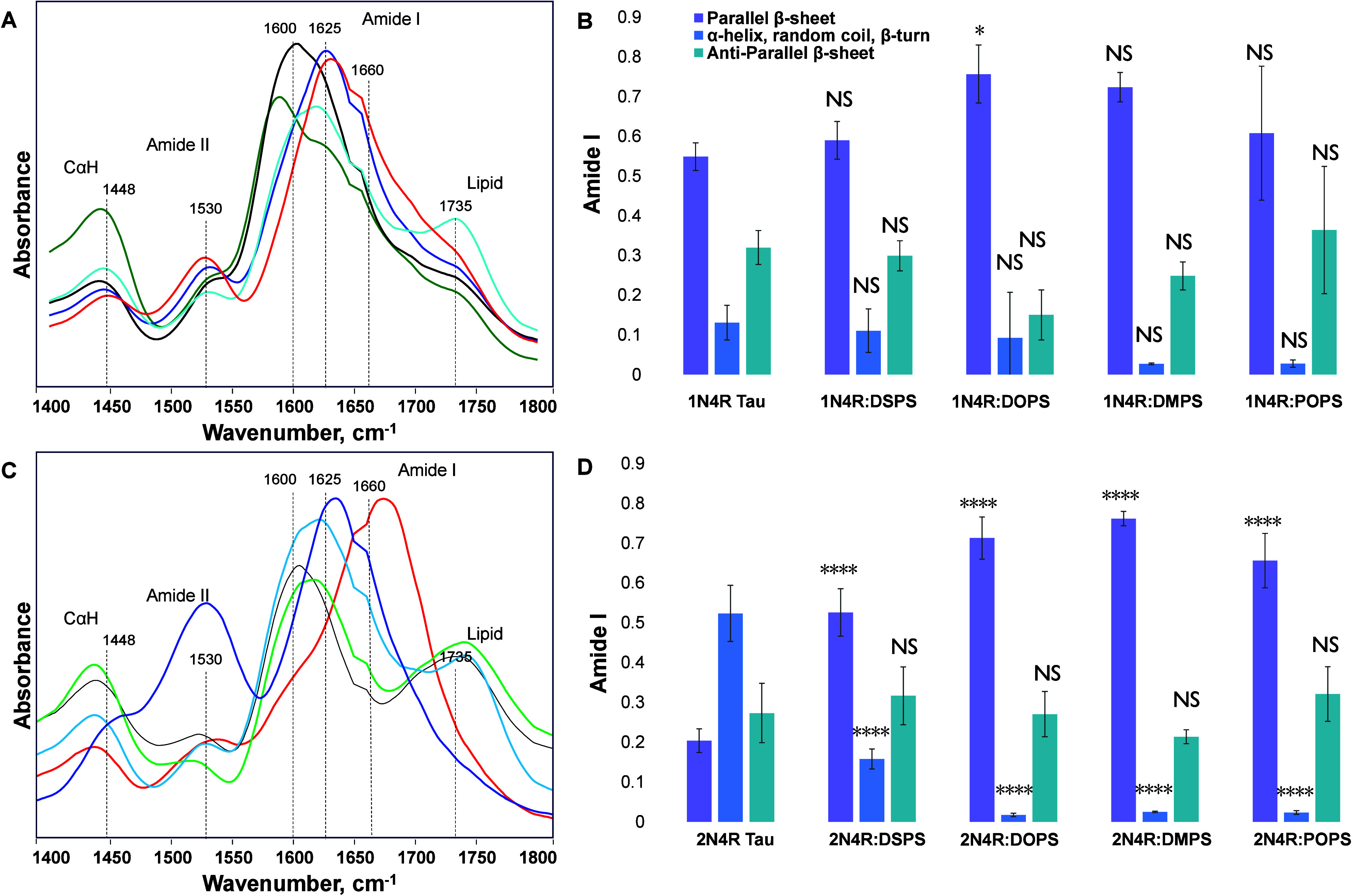
AFM-IR spectra (A and C) acquired from 1N4R Tau (A and B) and 2N4R Tau (C and D) aggregates with corresponding fitting of amide I band (B and D) indicating the amount of parallel β-sheet (purple) and α-helix, random coil and β-turn (blue) as well as antiparallel β-sheet (marine). According to one-way ANOVA, NS is nonsignificant difference, *P < 0.05, and ****P < 0.0001.

AFM-IR revealed that the secondary structure of 2N4R Tau fibrils grown in the lipid-free environment was dominated by unordered protein secondary structure (∼50%), with a small amount of parallel (∼20%) and antiparallel (∼30%) β-sheet, Figure 4C,D. We observed a drastic increase in the amount of parallel β-sheet in all 2N4R Tau aggregates that were formed in the presence of PS. It should be noted that oligomers detected in 2N4R:DOPS, 2N4R:DMPS, and 2N4R:POPS were nearly entirely composed of parallel β-sheet with a very small amount of unordered protein secondary structure and antiparallel β-sheet. These results indicate that PS with both saturated and unsaturated FAs drastically altered the secondary structure of 2N4R Tau aggregates.

Lactate dehydrogenase (LDH)-based toxicity analysis of 1N4R Tau fibrils formed in the heparin- and lipid-free environment revealed their high cytotoxicity, Figure 5. Similar cytotoxicity was also observed for 1N4R:DSPS fibrils. At the same time, we found that β-sheet-rich oligomers formed in the presence of POPS, DOPS, and DMPS exerted significantly lower cytotoxicity to N27 rat dopaminergic cells compared to 1N4R Tau and 1N4R:DSPS fibrils. These results indicate that PS with short (DMPS) and unsaturated FAs (POPS and DOPS) drastically lowers the cytotoxicity of 1N4R Tau fibrils.

**Figure 5:**
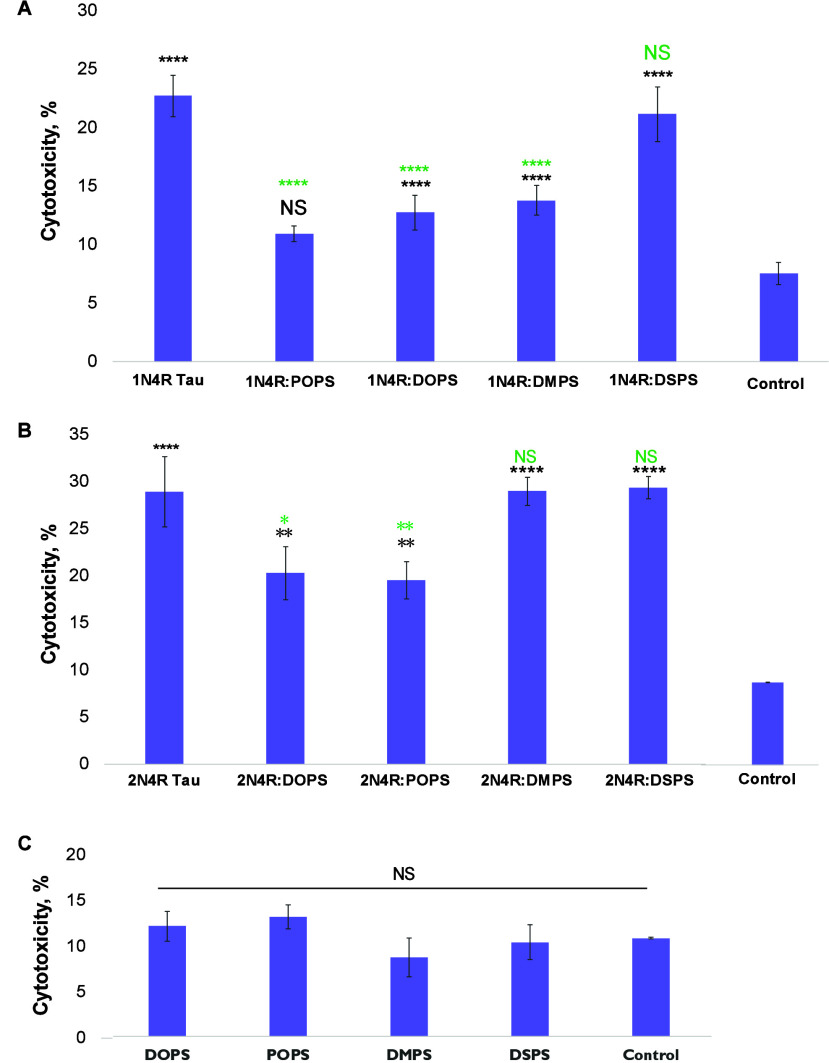
Histograms of LDH assay showing cytotoxicity of 1N4R (A) and 2N4R Tau (B) aggregates formed in the heparin-free environment with and without lipids as well as cytotoxicity (C) of lipids themselves. Black asterisks (*) indicate statistical significance of all samples relative to control. Green asterisks (*) indicate statistical significance of protein aggregates formed in the presence of lipids relative to the aggregates formed in the lipid-free environment. According to one-way ANOVA, NS is nonsignificant difference, *P < 0.05, **P < 0.01, ***P < 0.001, and ****P < 0.0001.

LDH assay revealed that 1N4R Tau and 2N4R Tau fibrils exerted similar levels of cell cytotoxicity. We also found that 2N4R:DOPS and 2N4R:POPS oligomers had significantly lower cytotoxicity compared with 2N4R Tau fibrils. Finally, the presence of DMPS and DSPS did not lower the cytotoxicity of the 2N4R Tau aggregates formed in their presence. These findings show that PS with unsaturated FAs (POPS and DOPS) drastically lowers the cytotoxicity of 2N4R Tau fibrils, while this effect was not evident for PS with short FAs (DMPS).

In summary, our results show that saturation and length of FAs in PS play an important role in aggregation of 1N4R and 2N4R Tau isoforms. We found that short-chain and unsaturated FAs in PS strongly inhibit fibril formation. Their equimolar presence with both 1N4R and 2N4R Tau results in the formation of small β-sheet-rich oligomers that exert significantly lower cytotoxicity to N27 neurons. At the same time, the presence of PS with long-chain saturated FAs alters the aggregation rate of only the 1N4R and 2N4R Tau isoforms. Such lipids alter the secondary structure of only 2N4R Tau oligomers, whereas no changes in the secondary structure were observed for 1N4R Tau aggregates that were formed in the presence of DSPS. Finally, both 1N4R:DSPS and 2N4R:DSPS fibrils exert the same cytotoxicity to N27 neurons as 1N4R and 2N4R Tau fibrils formed in the lipid-free environment. Thus, we can conclude that saturation and length of FAs in PS that are present at the stage of 1N4R and 2N4R Tau aggregation strongly alter the cytotoxicity of amyloid aggregates.

## Supporting Information Available

Materials and methods; CD spectra; AFM images; FTIR and AFM-IR spectra (PDF)
