# Supplementary material for: Under Heparin-Free Conditions Unsaturated Phospholipids Inhibit the Aggregation of 1N4R and 2N4R Tau
Source: J Phys Chem Lett. 2024 Aug 14;15(33):8577–83. doi: 10.1021/acs.jpclett.4c01718 (PMC11345945; doi:10.1021/acs.jpclett.4c01718)
Supplement: Supplementary file 1 — jz4c01718_si_001.pdf [file jz4c01718_si_001.pdf]

Under Heparin-Free Conditions Unsaturated Phospholipids Inhibit the Aggregation of 1N4R and 2N4R Tau

Abid Ali,[1] Mikhail Matveyenka,[1] Axell Rodriguez,[1] and Dmitry Kurouski*[1,2]

1. Department of Biochemistry and Biophysics, Texas A&M University, College Station, Texas 77843, United States
2. Department of Biomedical Engineering, Texas A&M University, College Station, Texas, 77843, United States

Email: dkurouski@tamu.edu

Supporting Information

Methods:

Materials: 1,2-dioleoyl-sn-glycero-3-phospho-L-serine (DOPS), 1-palmitoyl-2-oleoyl-sn-glycero-3-phospho-L-serine (POPS), 1,2-dimyristoyl-sn-glycero-3-phospho-L-serine (DMPS) and 1,2-distearyl-*sn*-glycero-3-phospho-L-serine (DSPS) were purchased from Avanti (Alabaster, AL, USA).

Liposome preparation: To prepare large unilamellar vesicles (LUVs) of DMPS, POPS, DOPS, and DPSP, 0.6 mg powder of the lipid was dissolved in 2.6 ml of phosphate-buffered saline (PBS) at pH 7.4. The solutions were then heated in a water bath at 50°C for 30 minutes. Subsequently, the solutions were rapidly immersed into liquid nitrogen for 3-5 minutes. This heating-thawing cycle was repeated 10 times. Finally, the lipid solutions were extruded 15 times through a 100 nm membrane using an extruder (Avanti, Alabaster, AL, USA). The LUV sizes (ranging from 90 to 100 nm) were determined using dynamic light scattering.er (Avanti, Alabaster, AL, USA). LUV sizes (90-100nm) were determined by dynamic light scattering.

Protein expression and purification Tau 2N4R and 1N4R: The proteins pET28B-Tau2N4R and pET28B-Tau1N4R were produced in Escherichia coli BL21 (DE3) Rosetta strain using LB broth media. A total of eight (8) liters of bacterial culture, with an optical density (OD) reading of 0.9, was induced with 1 mM Isopropyl β-D-1-thiogalactopyranoside (IPTG) and allowed to incubate at 16°C overnight. After the desired induction period, the overnight cultures were centrifuged at 8,000 RPM for 10 minutes to form a pellet. This pellet was then re-suspended in lysis buffer consisting of 8M Urea, 50 mM Tris-HCl, 300 mM NaCl, pH 8.0, supplemented with a protease inhibitor cocktail containing 1 mM Phenylmethylsulfonyl fluoride (PMSF). The re-suspended culture underwent 5 cycles of freeze-thaw followed by sonication at 28% Amplitude with 30 seconds on and off. Following sonication, the samples were centrifuged at 16,000g for 1 hour to separate the soluble fraction, and the resulting supernatants were carefully collected. To remove any particulate matter, the supernatants were passed through a syringe filter with a pore size of 0.4 µm. The filtered supernatants were then subjected to affinity chromatography using Ni-NTA agarose beads within a gravity column setup. Before elution, the column was extensively washed with a buffer containing 50 mM Tris-HCl, 300 mM NaCl, and 20 mM Imidazole to remove non-specifically bound proteins. The target proteins were subsequently eluted from the column using an elution buffer containing 50 mM Tris-HCl, 300 mM NaCl, and 300 mM Imidazole, in a total volume of 50 ml. The eluted protein fractions were dialyzed against PBS (pH 7.4) using a dialysis membrane with a molecular weight cut-off of 30 kDa to remove urea and imidazole. After dialysis, protein samples were concentrated using centrifugal concentrators with a 10 kDa molecular weight cut-off. The final concentration of the purified protein was approximately 2.5 mg/ml. Sodium Dodecyl Sulfate Polyacrylamide Gel Electrophoresis (SDS-PAGE) analysis was conducted to assess the purity and size of the obtained proteins.

2N4R and 1N4R Tau aggregation: In a lipid-free environment, 30 μM of protein was in PBS, and the solution pH was adjusted to pH 7.4. For the aggregation study of 2N4R and 1N4R Tau in the presence of phosphatidylserine (PS), 30 μM of protein was mixed with a (1:5) ratio of the concentration of the corresponding large unilamellar vesicles (LUVs). The pH of the final solution was adjusted to pH 7.4 using concentrated HCl. Subsequently, the samples were dispensed into a 96-well plate, which was placed in a plate reader (Tecan, Männedorf, Switzerland) at 37°C for 150 h under 510 rpm agitation..

Kinetic measurements: Rates of protein aggregation were measured using thioflavin T (ThT) fluorescence assay. For this, samples were mixed with 2 mM of ThT solution and placed into 96 well-plate that was kept in the plate reader (Tecan, Männedorf, Switzerland) at 37 °C for 150 h under 510 rpm agitation. Fluorescence measurements were taken every 10 min (excitation 450 nm; emission 488 nm). Each kinetic curve is an average of four independent measurements.

Atomic force microscopy (AFM) imaging: We utilized an AIST-NT-HORIBA system (Edison, NJ) AFM setup for the morphological analysis of protein aggregates. Silicon tapping-mode AFM probes from Appnano (Mountain View, CA, USA) were employed, featuring a force constant of 2.7 N/m and a resonance frequency of 50-80 kHz. The probe's diameter was 10 nm. To prepare for AFM imaging, each sample aliquot was diluted with DI water and deposited onto pre-cleaned glass coverslips. After exposure for 20-30 minutes, excess solution was removed from the glass surface, and the coverslips were dried using a flow of dried nitrogen. Approximately 20-30 individual aggregates were measured for each sample. Post-acquisition processing of the AFM images was performed using AIST-NT software (Edison, NJ, USA). We report their heights only because height measurements are absolute (artifact-free) in AFM, whereas widths have tip-convolution error and, therefore, should not be reported because they do not provide accurate information about the width of aggregates.

Attenuated total reflectance Fourier-transform Infrared (ATR-FTIR) spectroscopy: After 150 h of incubation at 37 °C, 2N4R and 1N4R samples were placed onto ATR crystal of 100 FTIR spectrometer (Perkin-Elmer, Waltham, MA, USA) and dried at room temperature. Three spectra were collected from each sample.

Atomic force microscopy-infrared spectroscopy (AFM-IR): Imaging and spectral analysis were obtained using a nanoIR3 system (Bruker, Santa Barbara, CA, USA), equipped with a QCL laser. ContGB-G AFM probes specific to contact mode were utilized with probe parameters: frequency of 13 kHZ, spring constant of 0.2 N/m, and a length of 450 μm. The tip was optimized using a polymethyl acrylate standard for the wavenumbers: 1400-1800 cm^{-1}. Laser parameters include a power of 25.49%, polarization at 90 degrees, IR focus of 70456, and a pulse rate around 828 kHz. Image dimensions of 1-10 μm were acquired at a scan rate of 0.5-0.8 Hz, an I and P gain ranging from 1/2 to 5/10, and a resolution of 256 for both the X and Y. A total of 30 spectra per sample were obtained with a co-average of 3 for each spectrum at a spectral resolution of 2 cm^{-1}/pt. In each sample, ~10 individual aggregates were analyzed. Protein samples were first deposited onto a 70 nm gold-coated silicon wafer at a volume of 2.5 μL, left to dry at room temperature for roughly 15 minutes or until a visible coffee ring under the drop is present, then rinsed with distilled water and dried using a N_2 air flow. The spectra were zapped at the 1648-1652 points to remove an artifact caused by the chip-to-chip transition of the laser at this region. Spectral processing was conducted using MATLAB, equipped with a PLS Toolbox version 9.0 (Eigenvector Research, Inc., Manson, WA). Spectra are first applied a smoothing processing of Savitzky-Golay at a polynomial order of 0, area normalized, and baselined with automated weighted least squares. After processing, spectra are then peak fitted using GRAMS/AI Spectroscopy Software to assign peaks respective to the secondary structure of the protein. Estimated area for each secondary structure is then run under ANOVA by first applying an Anderson-Darling Test to determine normal distribution, if so, a one-way ANOVA with Tukey HSD post-hoc test is run to determine statistical significance.

Cell toxicity assays: Rat midbrain dopaminergic N27 cells were cultured in RPMI 1640 Medium (Thermo Fisher Scientific, Waltham, MA, USA) supplemented with 10% fetal bovine serum (FBS) (Invitrogen, Waltham, MA, USA) in 96-well plates at a seeding density of 10,000 cells per well. The cells were then incubated at 37°C in a humidified atmosphere with 5% CO_2. After 24 hours, the cells had adhered fully to the culture surface.

Subsequently, 100 μL of the cell culture medium was replaced with 100 μL of RPMI 1640 Medium containing 5% FBS and supplemented with 10 μL of 2N4R and 1N4R protein aggregates. Following 24 hours of incubation with the protein aggregates, the toxicity of the aggregates was assessed using a lactate dehydrogenase (LDH) assay kit (G1781, Promega, Madison, WI, USA). Absorbance measurements were taken at 490 nm using a plate reader (Tecan, Männedorf, Switzerland).

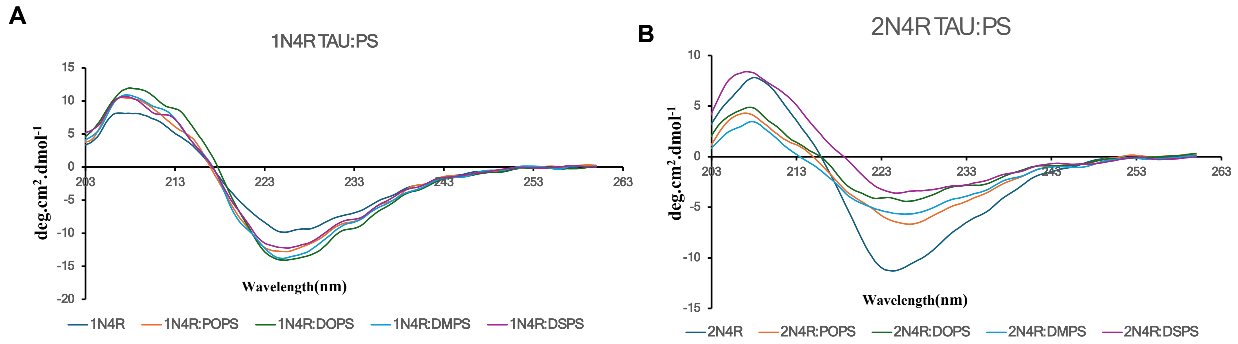

Figure S1. CD spectra acquired from 1N4R Tau (A) and 2N4R Tau (B) fibrils grown in the lipid-free environment and in the presence of lipids.

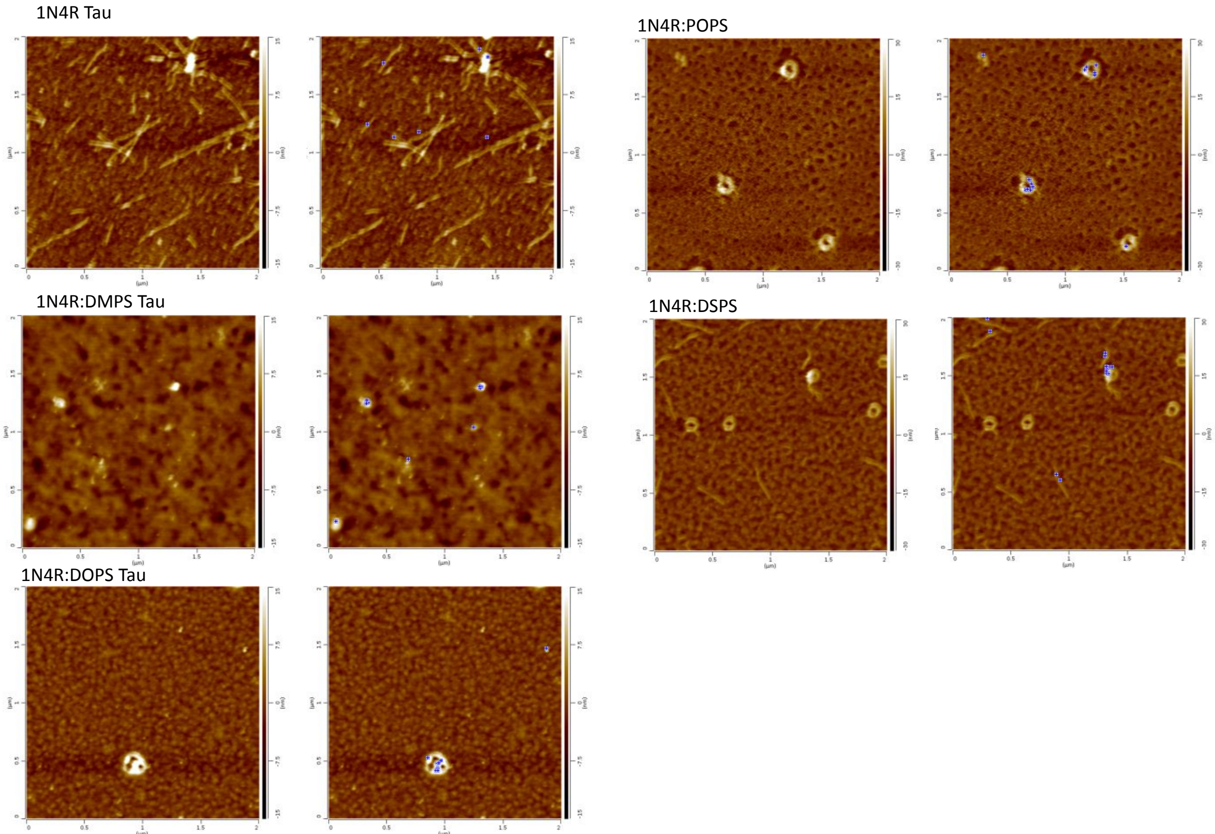

Figure S2. Representative AFM images of 1N4R aggregates (left panels) AFM images with sites of AFM-IR spectral acquisition (blue dots) (right panels).

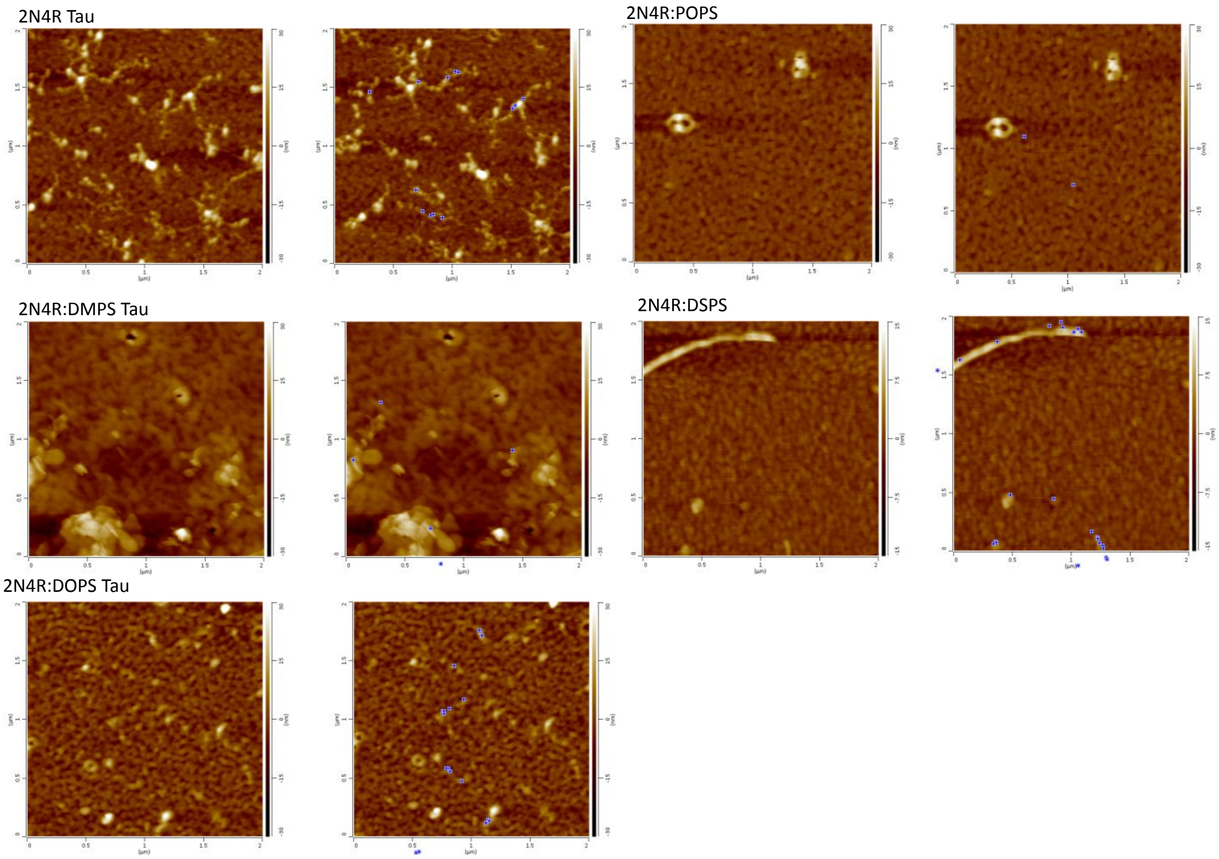

Figure S3. Representative AFM images of 2N4R aggregates (left panels) AFM images with sites of AFM-IR spectral acquisition (blue dots) (right panels).

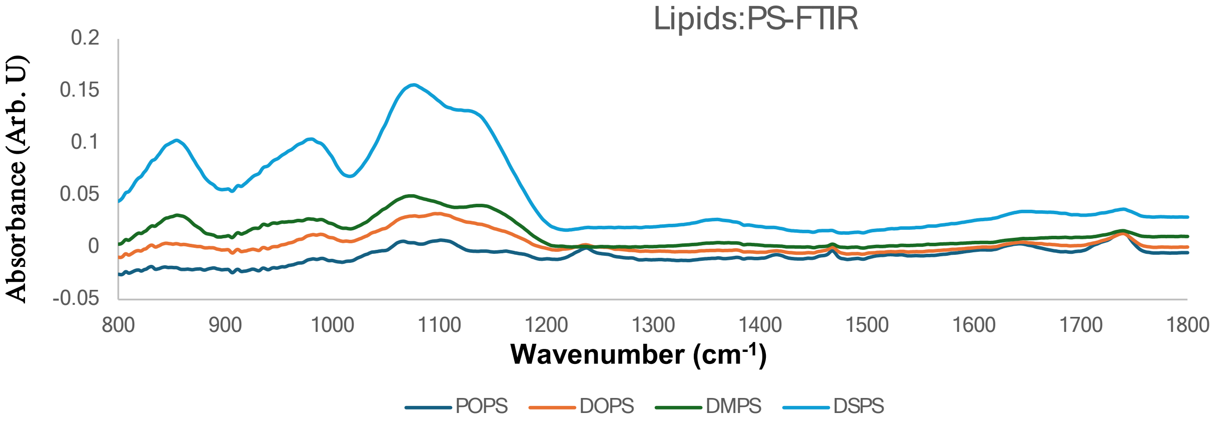

Figure S4. FTIR spectra of phospholipids.

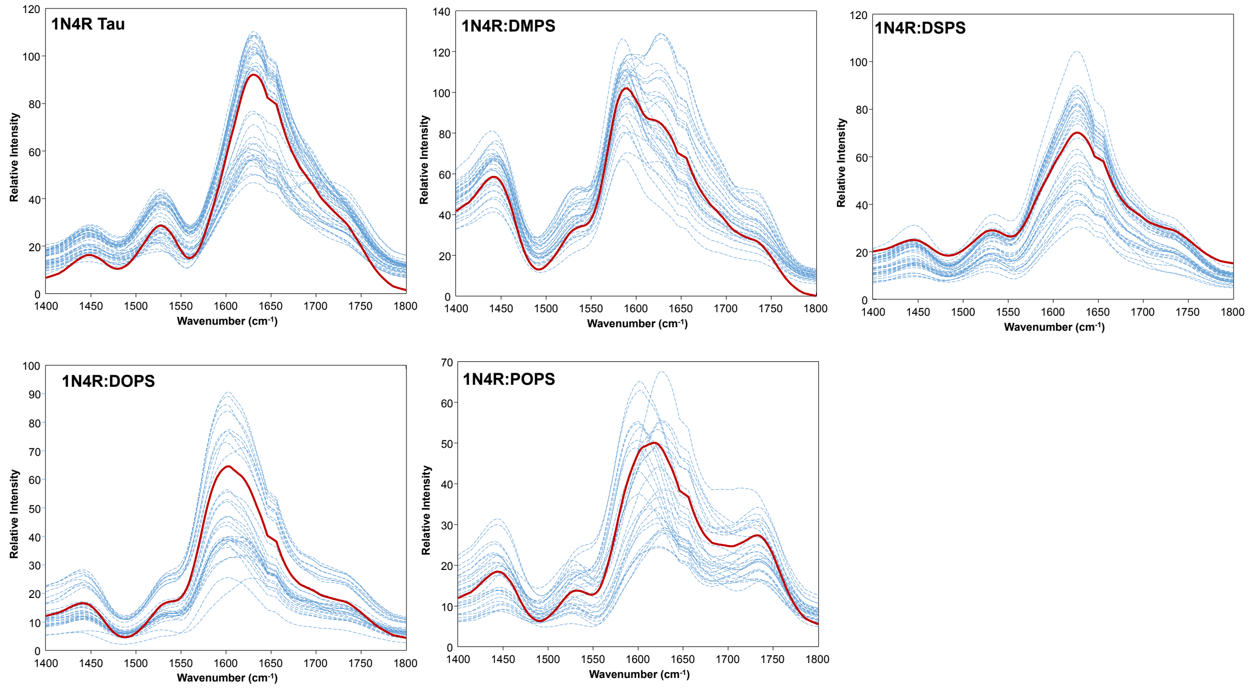

Figure S5. Individual (dotted blue) and averaged (Red) AFM-IR spectra acquired from 1N4R aggregates.

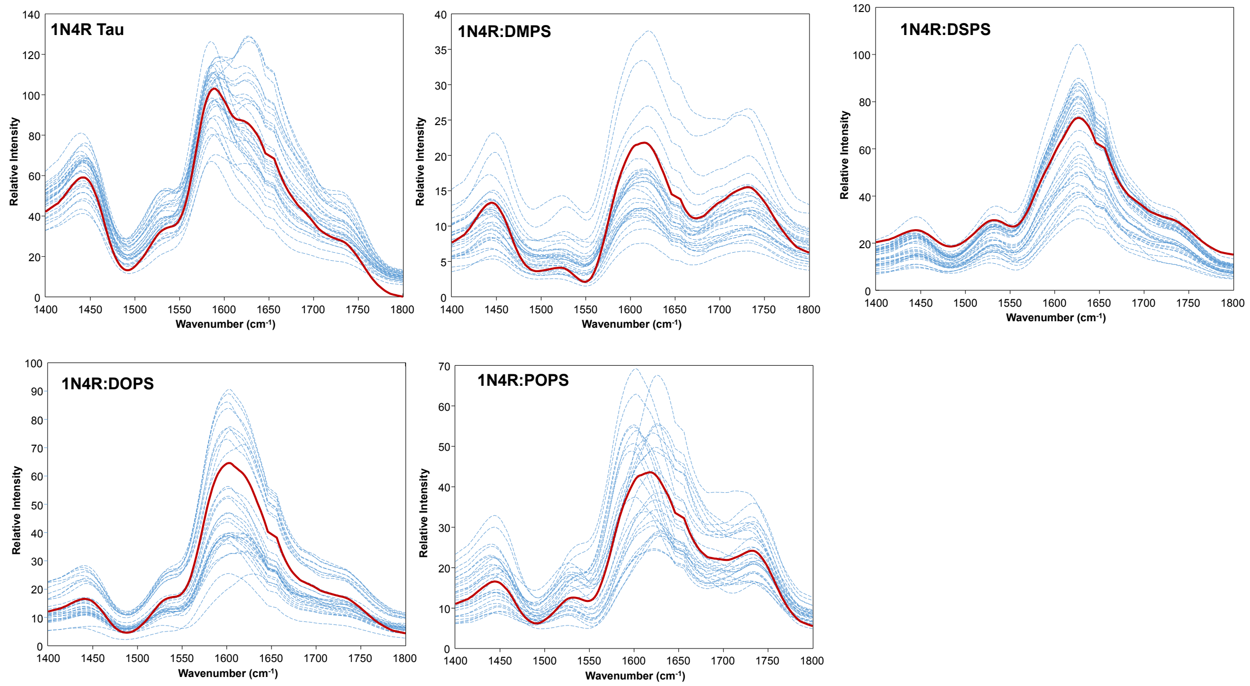

Figure S6. Individual (dotted blue) and averaged (Red) AFM-IR spectra acquired from 2N4R aggregates.
